# Early life manganese exposure and reported attention-related behaviors in Italian adolescents

**DOI:** 10.1097/EE9.0000000000000274

**Published:** 2023-10-19

**Authors:** Samantha Schildroth, Julia Anglen Bauer, Alexa Friedman, Christine Austin, Brent A. Coull, Donatella Placidi, Roberta F. White, Donald Smith, Robert O. Wright, Roberto G. Lucchini, Manish Arora, Megan Horton, Birgit Claus Henn

**Affiliations:** a Department of Environmental Health, Boston University School of Public Health, Boston, Massachusetts; b Department of Epidemiology, Geisel School of Medicine, Dartmouth College, Lebanon, New Hampshire; c Department of Environmental Medicine and Public Health, Icahn School of Medicine at Mount Sinai, New York City, New York; d Department of Biostatistics, Harvard T. H. Chan School of Public Health, Boston, Massachusetts; e Department of Occupational Health, University of Brescia, Brescia, Italy; f Department of Neurology, Boston University, Boston, Massachusetts; g Department of Microbiology and Environmental Toxicology, University of California Santa Cruz, Santa Cruz, California; h Department of Pediatrics, Icahn School of Medicine at Mount Sinai, New York City, New York; i Department of Environmental Health Sciences, Florida International University, Miami, Florida

**Keywords:** Attention, Critical periods, Manganese, Metals, Neurodevelopment, Teeth

## Abstract

**Background:**

Manganese (Mn) is an essential nutrient and neurotoxicant, and the neurodevelopmental effects of Mn may depend on exposure timing. Less research has quantitatively compared the impact of Mn exposure on neurodevelopment across exposure periods.

**Methods:**

We used data from 125 Italian adolescents (10–14 years) from the Public Health Impact of Metals Exposure Study to estimate prospective associations of Mn in three early life exposure periods with adolescent attention-related behaviors. Mn was quantified in deciduous teeth using laser ablation-inductively coupled plasma-mass spectrometry to represent prenatal (2nd trimester-birth), postnatal (birth ~1.5 years), and childhood (~1.5–6 years) exposure. Attention-related behavior was evaluated using the Conners Behavior Rating Scales in adolescence. We used multivariable linear regression models to quantify associations between Mn in each exposure period, and multiple informant models to compare associations across exposure periods.

**Results:**

Median tooth Mn levels (normalized to calcium) were 0.4 area under the curve (AUC) ^55^Mn:^43^Ca × 10^4^, 0.1 AUC ^55^Mn:^43^Ca × 10^4^, and 0.0006 ^55^Mn:^43^Ca for the prenatal, postnatal, and childhood periods. A doubling in prenatal tooth Mn levels was associated with 5.3% (95% confidence intervals [CI] = −10.3%, 0.0%) lower (i.e., better) teacher-reported inattention scores, whereas a doubling in postnatal tooth Mn levels was associated with 4.5% (95% CI = −9.3%, 0.6%) and 4.6% (95% CI = −9.5%, 0.6%) lower parent-reported inattention and attention deficit/hyperactivity disorder index scores, respectively. Childhood Mn was not beneficially associated with reported attention-related behaviors.

**Conclusion:**

Protective associations in the prenatal and postnatal periods suggest Mn is beneficial for attention-related behavior, but not in the childhood period.

**What this study adds**

This study is among the first to report manganese (Mn)-neurodevelopment associations in multiple exposure periods, where Mn exposure in the prenatal and postnatal periods was beneficially associated with adolescent attention-related behaviors. Conversely, Mn exposure in the childhood period was not beneficially associated with neurodevelopment. Our study supports the hypothesis that Mn neurotoxicity in early life is dependent on the timing of exposure.

## Introduction

Manganese (Mn) is a metal required for normal biological functions in humans.^1^ Mn is a component of various enzymes that are involved in neurodevelopment, making it an essential nutrient for neurodevelopment.^2^ However, overexposure to Mn, especially in populations exposed to anthropogenic sources like air pollution, has been associated with neurotoxicity in both human and animal models.^3–5^ Emerging epidemiological evidence suggests that the degree to which Mn acts beneficially versus as a neurotoxicant may depend not only on dose but also on the timing of exposure.^6^

Mn exposure in the prenatal period (~2nd trimester to birth) has been beneficially associated with cognition, including decreased scores for externalizing symptoms (e.g., aggression, hyperactivity) in some epidemiologic studies.^6,7^ This likely reflects the role of Mn as an essential nutrient during pregnancy.^7,8^ Fetal exposure to Mn is tightly regulated during gestation, and Mn is actively transported across the placenta to support the growth and development of the fetus.^9,10^ Further, Mn superoxide dismutase enzymes are critical for protecting the placenta and the developing fetus from oxidative stress.^11^ However, other studies have reported nonlinear or adverse associations of prenatal Mn with neurodevelopment,^12–15^ suggesting the dose of environmental exposure during this period of development plays a critical role in cognitive outcomes in children.

Mn exposure in the early postnatal period and during childhood has been more consistently associated with worse attention-related behaviors, including attention deficit/hyperactivity disorder (ADHD), internalizing problems, and externalizing symptoms, such as hyperactivity and oppositional behavior.^7,16–29^ Neurotoxicity resulting from early life Mn exposure has been observed in animal models,^30–33^ and reflects the toxic mechanisms of Mn in the brain, including induction of oxidative stress and inflammation, mitochondrial disruption of neurons, and disruption of neurotransmission.^34^ Greater evidence points to Mn toxicity in the postnatal and childhood periods, as compared with the prenatal period, which may be due to differences in dose, but also to differences in exposure sources and behavioral factors across developmental periods. For example, whereas fetal exposure to Mn in the prenatal period is regulated by the placenta,^10^ infants and children are exposed to environmental Mn through a variety of sources, including contaminated dust, air emissions, infant formula, and other dietary sources.^35^ Hand-to-mouth behavior in early childhood may also increase Mn exposure from the environment via ingestion, and consequent gastrointestinal absorption of Mn tends to be higher in young children compared with adults.^35^ The toxicity of Mn in early life may also reflect susceptibility to neurotoxic insults affecting the prefrontal cortex, which modulates attention, executive function, and emotion regulation during these developmental periods.^36^ This hypothesis is supported by animal data, where early life Mn exposure in rats led to altered concentrations of the neurotransmitters serotonin, dopamine, and norepinephrine, and their receptors, in the prefrontal cortex.^30,32,33^

Despite prior findings describing differential associations of Mn with neurodevelopment in the prenatal, postnatal, and childhood periods, less research has quantitatively compared Mn neurotoxicity across exposure periods, especially for attention-related behaviors. Attention, formally defined as the ability to process incoming information,^37^ is critical for the overall cognitive performance of children: attentional deficits have been linked with worse scholastic performance, including reading speed and accuracy.^38,39^ Scholastic performance, in turn, plays a role in cognitive function later in life.^40,41^ Therefore, quantifying the impact of early life Mn on attention-related behavior is of public health interest. The goal of the current analysis was to identify critical periods of Mn exposure (prenatal, postnatal, early childhood) measured in deciduous teeth in relation to reported attention-related behaviors in adolescence. Given emerging evidence of sexual dimorphism in Mn-neurodevelopment associations,^15,42–44^ we also explored potential sex differences in associations between Mn and Conners scores in each early life period.

## Methods

### Study population

We used cross-sectional data from the Public Health Impact of Metals Exposure (PHIME) study, which was designed to examine associations between metals exposure from ferroalloy industry emissions and neurodevelopment in early adolescence. A total of 721 adolescents (10–14 years) were recruited from three regions in the province of Brescia in northern Italy, including Bagnolo Mella (BM), Garda Lake (GL), and Valcamonica (VC). These three regions each had varied historical ferroalloy industry: BM has had active ferroalloy industry since 1974, GL had no ferroalloy industry, and VC had a ferroalloy industry that ceased in 2001.

Adolescents were recruited in two distinct phases that reflected two waves of funding for the study: the first phase (2007–2010) enrolled 311 adolescents and the second phase (2010–2014) enrolled 410 adolescents. All study protocols and questionnaires were consistent between the study phases. The second phase specifically recruited adolescents from the BM site and administered the Home Observation Measurement of the Environment (HOME) Short Form.^45^ Participants were eligible for enrollment if they were (1) 10–14 years of age at the time of recruitment, (2) had lived in the study area since birth, and (3) were born into families who resided in the study region since the 1970s. Participants were excluded from the study if they (1) had a diagnosed neurologic, psychiatric, hepatic, endocrine, or metabolic disease, (2) had clinically relevant motor deficits that could have impacted testing, (3) used medication with any neurologic side effects, (4) had clinically diagnosed behavioral or cognitive impairments, (5) had vision deficits with no corrective measures, or (6) had ever received parenteral nutrition. PHIME study procedures have been described in depth previously in the literature.^46^

**Figure 1.**
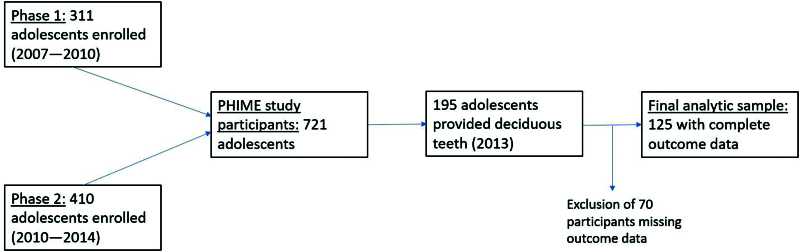
Schematic of the Public Health Impact of Metals Exposure (PHIME) study.

Additional supplemental funding was received in 2013 to collect deciduous teeth and quantify Mn levels from a subset of the PHIME participants (n = 195). The current analytic sample comprised 125 adolescents who provided teeth and had complete outcome data (Figure 1).

Informed consent was given by all participants after receiving detailed study information. Study protocols were approved by human subjects review boards (Institutional Review Boards) at the Icahn School of Medicine at Mount Sinai, University of California Santa Cruz, and the Ethical Committee of Brescia.

### Tooth collection and manganese measurement

We collected and measured Mn levels in one naturally shed deciduous tooth (incisor, canine, or molar) that was absent of obvious decay (i.e., caries) per child. Upon collection, each tooth was assessed for loss of dentin layers due to natural wear (i.e., attrition), which can affect the accuracy of Mn measurements.^6^ Tooth attrition was defined as an ordinal variable reflecting tooth tissue loss due to natural wear as follows: no tissue loss, less than one-third tissue loss, or one-third or more but less than two-thirds tissue loss. Teeth with two-thirds or more tissue loss were not analyzed.

All tooth analysis protocols have been described in detail elsewhere.^47,48^ In brief, a full cross-section of dentin for each tooth was exposed by sectioning teeth vertically. The neonatal line (NL), a histological feature that forms within the tooth at birth, was identified for each tooth.^49^ Using the NL as a temporal reference, a total of forty measurements were taken from the primary (n = 30) and secondary (n = 10) dentin to estimate Mn levels in three exposure periods: prenatal, measured in primary dentin and reflecting Mn exposure from the second trimester to birth; early postnatal, measured in primary dentin and reflecting Mn exposure from birth to ~1.5 years; and childhood, measured in secondary dentin and reflecting exposure from ~1.5 to 6 years or until the tooth is shed. Mn levels were quantified using laser ablation-inductively coupled plasma-mass spectrometry.^47,48^ Tooth calcium (Ca) levels were analyzed using the same methods as tooth Mn. All tooth Mn levels were normalized to tooth Ca levels as the ratio of Mn to Ca (^55^Mn:^43^Ca, unitless) to account for differences in mineral density of the tooth within and between participants.

To reflect cumulative exposure in each period, prenatal and postnatal Mn levels were estimated as the ^55^Mn: ^43^Ca area under the curve (AUC) × 10,000 across all primary dentin sampling points; childhood ^55^Mn: ^43^Ca levels were averaged across secondary dentin measurements. Only two values in the postnatal period were below the limit of detection (LOD) of 0.03 ^55^Mn: ^43^Ca × 10,000; these values were imputed as the LOD/2. Laboratory technicians were blinded to participants’ neurodevelopmental outcomes.

### Cognitive assessment

We used the Conners Rating Scales (CRS) to assess attention-related behavior. The CRS is a validated neurodevelopmental assessment tool developed to determine clinically relevant ADHD symptomology in children.^50–52^ It is a comprehensive, multi-informant assessment with three scales for parent-, teacher-, and self-reports: Conners–Wells’ Adolescent Self-Report Scale-Long Form, S-Conners’ Parent Rating Scales Revised-Short Form, and S-Conners’ Teaching Rating Scale-Short Form. Scores from both the parent- and teacher-reports have been shown to have good internal consistency and long-term test-retest reliability;^53^ therefore, we focused our primary analyses on scores from parent- and teacher-reports. The parent- and teacher-reports include scales for oppositional behavior, inattention/cognitive problems, hyperactivity, and an ADHD index.^50^ The CRS was administered to participants in Italian by trained examiners who were blinded to the exposure status of participants.^53^ Raw scores were converted into standardized T-scores (mean: 50, standard deviation: 10) that were adjusted for age and sex.

### Measurement of confounders

Standardized questionnaires were administered either in person or via the phone by trained study staff. Information was collected on socioeconomic and demographic covariates, including age at enrollment (continuous, years), biological sex (male vs. female), area of residence (BM, GL, or VC), parental occupation, parental education, and tooth attrition. Nine items from the HOME Short Form were administered to participants, and scores ranged from 0 to 9.^45^ We classified each participant’s socioeconomic status (SES; low, medium, or high) based on parental education and occupation using a methodology developed for Italian populations, as described previously.^54,55^

Tooth lead (Pb) levels, reflecting Pb exposure in the same exposure periods as Mn, were not quantified. However, we measured Pb concentrations in whole blood collected in adolescence at the time of neurodevelopmental assessment. Whole blood samples (4 ml) were collected using 19-gauge butterfly catheters and stored in lithium-heparin Sarstedt Monovette Vacutainers. Concentrations of Pb were quantified using magnetic sector inductively coupled plasma-mass spectrometry.^46,56,57^ All blood Pb measurements were above the LOD (0.03 ng/ml).

### Statistical analysis

We first examined distributions of Conners scores, covariates, and tooth Mn levels in each of the three exposure periods. Conners scores, blood Pb, and tooth Mn levels were right-skewed; these variables were natural log (ln)-transformed to meet the assumption of normality of residuals and reduce the influence of extreme values. Summary statistics were calculated for all variables (Table 1) and we estimated Spearman correlation coefficients between Mn levels in the prenatal, postnatal, and childhood periods.

**Table 1.** Summary statistics for the PHIME study population

Characteristic	N (%) or mean (SD)	N (%) or mean (SD)	N (%) or mean (SD)
	Full cohort (n = 125)	Males (n = 57)	Females (n = 68)
Age (years)	11.9 (0.9)	11.8 (0.8)	12.0 (0.9)
Socioeconomic status index			
Low	26 (20.8%)	5 (8.8%)	21 (30.9%)
Medium	71 (56.8%)	40 (70.2%)	31 (45.6%)
High	28 (22.3%)	12 (21.0%)	16 (23.5%)
HOME score	6.3 (1.4)	6.3 (1.4)	6.3 (1.5)
Site			
Bagnolo Mella	74 (59.2%)	35 (61.4%)	39 (57.4%)
Garda Lake	28 (22.4%)	11 (19.3%)	17 (25.0%)
Valcamonica	23 (18.4%)	11 (19.3%)	12 (17.6%)
Tooth loss due to attrition			
None	68 (54.4%)	26 (45.6%)	42 (61.8%)
Less than one-third	46 (36.8%)	24 (42.1%)	22 (32.3%)
One-third or more but less than two-thirds	11 (8.8%)	7 (12.3%)	4 (5.9%)
Two-thirds or more	0 (0%)	0 (0%)	0 (0%)
Conners parent-reported T-scores			
ADHD index	50.1 (11.3)	48.7 (8.4)	51.3 (13.2)
Hyperactivity	48.1 (8.2)	47.3 (7.2)	48.8 (9.0)
Inattention	48.9 (10.8)	46.9 (8.5)	50.6 (12.3)
Oppositional behavior	49.1 (10.0)	48.9 (11.0)	49.1 (9.2)
Conners teacher-reported T-scores			
ADHD index	45.8 (6.4)	44.7 (7.7)	46.6 (5.1)
Hyperactivity	45.0 (5.1)	43.7 (6.3)	46.1 (3.4)
Inattention	47.5 (7.6)	46.7 (8.3)	48.1 (6.9)
Oppositional behavior	45.4 (4.3)	44.0 (4.0)	46.6 (4.2)
Metal biomarker: median (25th, 75th percentile)			
Tooth Mn, Prenatal (AUC ^55^Mn:^43^Ca × 10^4^)^a^	0.4 (0.3, 0.5)	0.4 (0.3, 0.5)	0.4 (0.4, 0.5)
Tooth Mn, Postnatal (AUC ^55^Mn:^43^Ca × 10^4^)^a^	0.1 (0.1, 0.2)	0.1 (0.1, 0.2)	0.1 (0.1, 0.2)
Tooth Mn, Childhood (average ^55^Mn:^43^Ca)^a^	0.0006 (0.0005, 0.0009)	0.0006 (0.0004, 0.0009)	0.0007 (0.0005, 0.0009)
Blood Pb (µg/dl)	1.6 (1.0, 1.8)	1.3 (1.1, 1.9)	1.3 (0.9, 1.5)

a prenatal period = 2nd trimester of gestation to birth, postnatal period = birth to ~1.5 years, childhood = ~1.5 years to 6 years.

ADHD indicates attention deficit hyperactivity disorder; AUC, area under curve; Ca, calcium; HOME, Home Observation Measurement of the Environment; Mn, manganese; Pb, lead.

Covariates were chosen a priori based on directed acyclic graphs and prior literature (eFigure 1; http://links.lww.com/EE/A243).^6,15,58^ All models were adjusted for SES, HOME score, ln-transformed blood Pb, and tooth attrition. Pb is a known neurotoxicant and may be associated with Mn exposure; we therefore hypothesized Pb to be a potential confounder of Mn-neurodevelopment associations among children residing near the ferroalloy industry.^59^ We did not include sex or age as covariates in statistical models because the Conners T-scores were age and sex adjusted. Additionally, all models were mutually adjusted for Mn levels in all three exposure periods (prenatal, postnatal, and early childhood).

Prior epidemiological evidence suggests that associations between Mn and neurodevelopment may be nonlinear because Mn is both an essential nutrient and a toxicant.^12,15,26,58^ We assessed the potential for nonlinear associations between Mn in each exposure period and Conners scores using covariate-adjusted generalized additive models with penalized splines (knots = 4). There was little evidence of nonlinearity based on visual inspection; therefore, Mn was modeled as a continuous variable in subsequent multivariable linear regression models.

There was little missing data (<7% missing for all variables; see eTable 1; http://links.lww.com/EE/A243), but to maximize the analytic sample and reduce potential bias,^60^ we used Monte Carlo Markov Chain multiple imputations to impute missing covariate data using the mice package in R,^61,62^ where data were assumed to be missing at random. We generated 20 imputed datasets for the full PHIME cohort (n = 721) using all variables possibly related to the missing data, including Mn levels in each exposure period, concentrations of all metals (Mn, Pb, copper, and chromium) measured in other biomarkers (hair, nail, saliva, blood, and urine),^63^ Mn concentrations in environmental samples (soil, air, water, and dust),^63^ Conners scores, and confounder data.^6^ We restricted our final analytic sample to adolescents with complete exposure and outcome data (n = 125), but used imputed values for missing confounder information.

Multivariable linear regression models were fit for each imputed dataset to examine the association between tooth Mn levels in the prenatal, postnatal, and childhood periods with each of the Conners scales while adjusting for a priori-determined confounders. We used Rubin’s rule to statistically combine findings from linear regression models across the 20 imputed datasets,^64^ and generated pooled beta coefficients and 95% confidence intervals (CI). For ease of interpretation, we back-transformed the beta coefficients and 95% CI to represent a percent change in Conners T-scores for a doubling in tooth Mn levels using the following equations:


$$\begin{array}{l} \%\;change\;in\;Conners\;scores=\left(e^{\left(ln\left(2\right)\;\times \;\beta \right)}\right)-1\times 100 \end{array}$$ (1)

$$\begin{array}{lll} & \%\;change\;in\;Conners\;scores,\;95\%\;CI=\; & \left(e^{\left(ln\left(2\right)\times \left(\beta \pm 1.96\times standard\;error\right)\right)}\right)-1\times 100 \end{array}$$ (2)

Next, we fit multiple informant models to test whether associations between Mn and Conners scores differed across the prenatal, postnatal, and childhood periods.^6,65^ A generalized estimating equation was fit for each Conners scale. Generalized estimating equations were first fit for each of the 20 imputed datasets and then findings were statistically combined using Rubin’s rule in SAS (for example code, see Bauer et al.^6^). Differences in associations of tooth Mn with Conners scales across exposure periods were considered significant based on a *P* < 0.10 from multiple informant models.

Emerging evidence suggests that associations between Mn and neurodevelopment may vary by biological sex.^15,16,42,44,66,67^ To explore potential sex differences in associations between Mn and Conners scores, we stratified our data by biological sex and fit multivariable linear regression and multiple informant models, as described above, in the stratified datasets.

All analyses were conducted in R version 3.6.1 and SAS version 9.4.

## Results

About half of the participants were female (54%), and most were from families that were classified as medium SES (57%) and lived in Bagnolo Mella (59%, Table 1). The mean age of participants with an available tooth for analysis was 11.9 years (standard deviation [SD]: 0.9 years) and the mean HOME score was 6.3 (SD: 1.4). The median blood lead concentration was 1.6 µg/dl (25th–75th percentile: 1.0, 1.8 µg/dl). Median tooth Mn levels (reported as AUC ^55^Mn: ^43^Ca × 10^4^) were higher in the prenatal period (0.4; 25th–75th percentile: 0.3, 0.5) than in the postnatal period (0.1; 25th–75th percentile: 0.1, 0.2). Correlations between Mn concentrations across the exposure periods were weak: −0.06 (prenatal-childhood), 0.07 (postnatal-childhood), and 0.27 (prenatal-postnatal). Median levels of Mn in the prenatal, postnatal, and childhood periods were similar between males and females (Table 1). Average Conners scores on the parent-reported scales were consistently higher (indicating more reported problem behaviors) than scores on teacher-reported scales. Conners scores within the same respondent were moderately to highly correlated (range, parent-reported: 0.41–0.81; teacher-reported: 0.31–0.65), whereas correlations for scores across respondents were weaker (0.09–0.53). Scores for both the parent- and teacher-reported scales tended to be higher in females compared with males (Table 1). Summary statistics were similar between imputed and complete data (eTable 2; http://links.lww.com/EE/A243).

**Figure 2.**
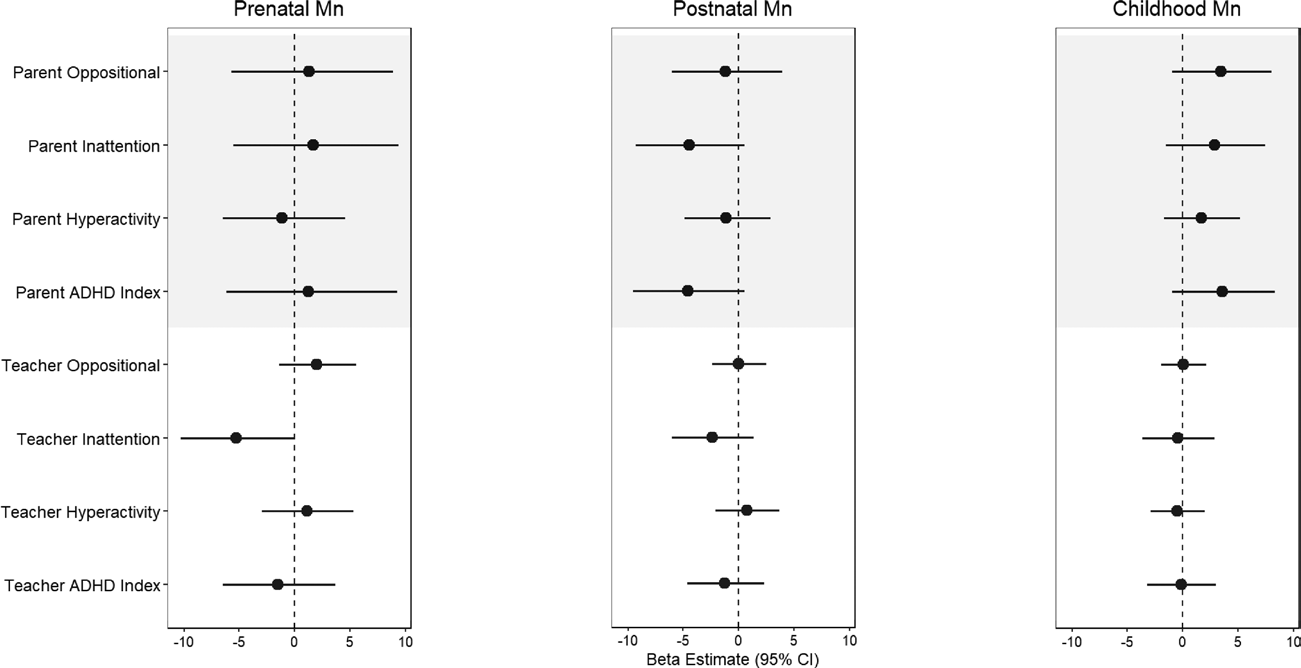
Adjusted beta (β) estimates and 95% CIs from multivariable linear regression models assessing associations between prenatal, postnatal, and childhood tooth Mn levels with parent- and teacher-reported scores from the Conners Rating Scales. Beta coefficients reflect the percent change in age- and sex-adjusted Conners T-scores for a doubling in tooth Mn levels. *Multivariable linear regression models were mutually adjusted for Mn in all exposure periods, and socioeconomic status, HOME score, tooth attrition, and ln-transformed blood Pb. **prenatal period = 2nd trimester of gestation to birth, postnatal period = birth to ~1.5 years, childhood = ~1.5 to 6 years.

In adjusted linear regression models, a doubling of prenatal tooth Mn levels was associated with 5.3% (95% CI = −10.3%, 0.0%) lower teacher-reported inattention T-scores, suggesting beneficial effects of prenatal Mn on attention-related behavior. This association was attenuated in the postnatal period and null in childhood (Figure 2, eTable 3; http://links.lww.com/EE/A243), and was significantly different across the three exposure periods in multiple informant models (*P* = 0.01, eTable 4; http://links.lww.com/EE/A243). Similar to the beneficial association observed in the prenatal period, a doubling in postnatal Mn levels was associated with lower T-scores for parent-reported inattention (β = −4.5%; 95% CI = −9.3%, 0.6%), parent-reported ADHD index (β = −4.6%; 95% CI = −9.5%, 0.6%), and teacher-reported inattention (β = −2.4%; 95% CI = −6.0%, 1.4%). Conversely, a doubling in childhood tooth Mn levels was associated with 2.9% (95% CI = −1.5%, −1.0%), 3.6% (95% CI = −0.9%, 8.4%), and 3.5% (95% CI = −0.9%, 8.1%) higher parent-reported inattention, ADHD index, and oppositional behavior scores, respectively (Figure 2), suggesting that childhood Mn levels were adversely associated with parent-reported attention-related behaviors. There was some evidence that associations with parent-reported inattention and ADHD index differed across exposure periods in multiple informant models, though the *P* values were ≥0.10 (*P* = 0.15 and *P* = 0.10, respectively).

**Figure 3.**
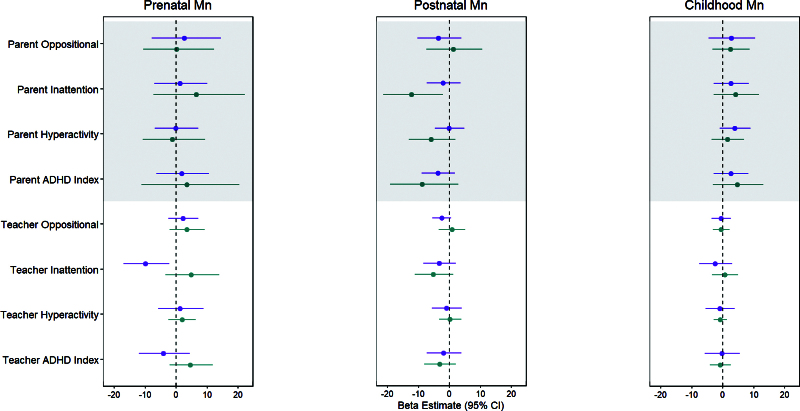
Adjusted beta (β) estimates and 95% CIs from sex-stratified multivariable linear regression models assessing associations between prenatal, postnatal, and childhood tooth Mn levels and change in age- and sex-adjusted Conners T-scores per doubling in tooth Mn levels. Females (n = 68) are shown in teal; males (n = 57) are shown in purple. *Multivariable linear regression models were mutually adjusted for Mn in all exposure periods, and socioeconomic status, HOME score, tooth attrition, and ln-transformed blood Pb. **prenatal period = 2nd trimester of gestation to birth, postnatal period = birth to ~1.5 years, childhood = ~1.5 to 6 years.

In exploratory sex-stratified models, a negative (i.e., beneficial) association between prenatal Mn and teacher-reported inattention was estimated in males (per doubling in Mn: β = −9.9%; 95% CI = −17.0%, −2.3%) but not in females (β = 4.8%; 95% CI = −3.5%, 13.9%, Figure 3). Based on multiple informant models, this beneficial association in the prenatal period among males differed significantly from the null associations in the postnatal and childhood periods (*P* < 0.01, eTable 5; http://links.lww.com/EE/A243).

Among females, negative (i.e., beneficial) associations between Mn and parent- and teacher-reported scales were also observed, but in the postnatal period: a doubling in postnatal Mn levels was associated with 12.3% (95% CI = −21.5%, −2.1%), 8.9% (95% CI = −19.2%, 2.8%), and 6.0% (95% CI = −13.2%, 1.9%) lower parent-reported T-scores for inattention, ADHD index, and hyperactivity, respectively, and 5.3% (95% CI = −11.2%, 1.1%) lower teacher-reported T-scores for inattention (Figure 3). Beneficial associations among females were not observed in the prenatal or childhood periods (Figure 3), although *P* values from multiple informant models comparing associations across exposure periods were not statistically significant (all *P* > 0.10, eTable 5; http://links.lww.com/EE/A243).

## Discussion

In this study of Italian adolescents, we found that prenatal Mn was associated with decreased T-scores for teacher-reported inattention and that this association was significantly different across exposure periods. Postnatal Mn was similarly associated with decreased scores for inattention and the ADHD index on parent-reported scales, whereas childhood Mn was associated with modest increases in scores for parent-reported scores. Overall, these findings support a beneficial association of Mn with reported attention-related behaviors when exposure occurred in the prenatal and postnatal periods, but not in the childhood period.

Consistent with the findings of the current analysis, we previously found protective associations between prenatal tooth Mn and scores on the Weschler Intelligence Scale for Children in the PHIME cohort.^6^ Beneficial associations between prenatal Mn and neurodevelopment have been observed in at least one other cohort: in Mexican children, prenatal tooth Mn was associated with decreased externalizing symptoms, aggression, and hyperactivity.^7^ Protective associations of early life Mn with neurodevelopment likely reflect the known need for Mn during prenatal development: physiological requirements for Mn increase during pregnancy to support fetal growth, and Mn is actively transported across the placenta.^68^ Specifically, Mn is essential for various enzymes used in neuronal tissues that mediate processes like protein synthesis and reactive oxygen species defense.^2^ On the other hand, our findings conflict with those of two other studies: Mora et al.^16^ found that prenatal tooth Mn in Mexican–American children was associated with increased internalizing problems, externalizing problems, and hyperactivity measured on the BASC at 10.5 years, and Skogheim et al.^8^ reported nonmonotonic dose responses for maternal (prenatal) blood Mn and attention-related behavior. These inconsistent findings may, in part, reflect differences in Mn exposure levels during pregnancy, the timing and test used to measure neurodevelopment, or SES across study populations. We were not able to directly compare the levels of tooth Mn in our study to those in other studies due to methodological differences in quantifying tooth Mn levels.^16^

Protective associations of tooth Mn with attention-related behaviors were also observed in the postnatal period, consistent with our prior work in PHIME.^6^ However, these findings conflict with findings from other cohorts, where postnatal tooth Mn was associated with increased internalizing problems in Mexican children,^7^ and with increased internalizing and externalizing problems among Mexican–American children.^16^ Evidence from animal models supports the notion that Mn exposure in the postnatal period may be neurotoxic, where early life Mn exposure in rats has been consistently associated with increased hyperactivity and attentional problems,^30–33^ and reductions in cortical norepinephrine, dopamine, and serotonin concentrations.^32^ Human brain maturation in the postnatal period is dynamic and occurs rapidly: in the first year of life, brain size increases from 36% (at birth) to 70% of the total adult brain size.^69^ This growth includes several maturation processes, such as synaptic remodeling and pruning, myelination, synapse formation, and axonal and dendritic outgrowth and branching.^69^ Thus, our findings may differ from prior studies in part because the exposure period that we captured for this highly dynamic and rapidly changing postnatal period (birth to ~1.5 years) was longer. This hypothesis is supported by findings from Horton et al.,^7^ where postnatal tooth Mn levels between 3 and 5 months were associated with reductions in externalizing symptoms in Mexican children, but this association was not present after 5 months. Future studies that examine postnatal Mn exposure with higher temporal resolution are warranted to identify possible critical periods of exposure within the early postnatal period.

Similar to our findings, Mn exposure in childhood has been consistently associated with worse attention-related behaviors and ADHD diagnosis.^21–23,28,70,71^ It should be noted that the timing of Mn exposure in these prior studies varied, and many studies included participants from both childhood (1–9 years) and adolescence (10–16 years).^17–26,28,70,71^ Adolescence is characterized by a rapid maturation of the prefrontal cortex (e.g., pruning) of the brain,^72^ suggesting susceptibility to Mn neurotoxicity may differ for this age group. Therefore, prior epidemiological findings may not be directly comparable to our population. Nonetheless, studies of early childhood have similarly found associations between Mn and worse attention-related behaviors. Two studies reported associations between water Mn exposure in the first 5 years of life and increased odds of ADHD diagnosis in children from Bangladesh and Denmark,^70,71^ whereas hair Mn quantified at 6–9 years was associated with worse performance on the Trail Making Test among Indonesian children.^23^ In a cross-sectional study of 5-year-old Chinese children, blood Mn was similarly associated with worse scores for hyperactivity, conduct problems, and the ADHD index on the CRS. This study also found that blood Mn was associated with decreased concentrations of the neurotransmitters glutamate and glycine, and that altered glycine concentrations mediated the association between Mn and increased Conners scores.^28^ These findings support the hypothesis that Mn neurotoxicity in children is due, in part, to interference with neurotransmission.

In exploratory analyses, there was evidence of sex-specific associations in all three exposure periods. In the prenatal period, tooth Mn was protective of reported inattention only in males. This is similar to findings from the CHAMACOS study of Mexican–American children, in which prenatal Mn was associated with decreased scores on the ADHD Confidence Index of the continuous performance test-II in males only.^16^ In the postnatal period, we found that Mn was also protective of parent-reported attention and hyperactivity, but in females. This was in contrast to findings from the CHAMACOS study, in which worse attention scores on the BASC were associated with Mn among females.^16^ Consistent with our findings, most studies of childhood Mn exposure, with the exception of one,^71^ reported no modification by sex.^25,26,29^ Sex-specific effects of Mn may be related to sex differences in the influence of Mn transporters on the regulation of Mn uptake across developmental periods.^66,67^

Although findings were consistent across parent- and teacher-reports for several scales (e.g., inattention), the magnitude and variability of associations differed between parent- and teacher-reports for other scales (e.g., ADHD index), whereby associations were observed with parent-reported but not teacher-reported, scores. This might reflect differences in reporting from parents versus teachers. Although scores on parent- and teacher-reports are moderately correlated (e.g., 0.64 for inattention) in normative samples,^73^ differences in parent- and teacher-reported scores have been reported in the literature.^73,74^ For example, one study in younger children reported higher (i.e., worse) average scores on the teacher than the parent report,^74^ whereas another among adolescents found that parents reported higher mean scores,^73^ which was the case in our study as well. Both prior studies concluded that differences in reported scores were likely the result of differing expectations in the home versus the school environment.^73,74^ In school, for example, children may be expected to have better attention and executive function to complete academic tasks. In adolescents, however, scores for teacher-report may be lower (i.e., better) than parental-report because individual teachers tend to spend less time with each student throughout the day than in childhood.^73^ Differences in reporting likely explain some of the differences in our findings across respondent types, though they do not inherently suggest a lack of validity. Instead, the findings of this study may simply reflect variations in attention-related behaviors for different environments.

It should further be noted that our findings, particularly for the prenatal period, were not consistent across all parent- and teacher-reported scales. Although there is evidence in the literature supporting the associations observed in the current analysis, we cannot rule out the possibility that these findings were spurious given our limited sample size. This small sample size reduced the statistical power and precision of effect estimates, in particular for the sex-stratified models, which should be considered exploratory. This study has additional limitations. Given the subjective nature of the CRS, it is likely that there is some degree of misclassification of the outcome metrics, though we would expect this error to be nondifferential with respect to exposure, and likely create a bias towards the null. We also had limited data on maternal characteristics in pregnancy and on participant characteristics in early life. For example, there may be residual confounding by maternal and early life iron status, given that iron is also an essential nutrient needed for neurodevelopment,^75^ and altered iron status (e.g., deficiency) has been associated with increased Mn biomarker concentrations.^76^ Finally, although we were able to adjust for adolescent blood Pb concentrations, we did not have early life measurements of Pb exposure or other toxic metals, which may have led to further residual confounding.

Our study also has several strengths. We used a tooth biomarker to retrospectively characterize Mn exposure in multiple developmental periods. Although the granularity with which we measured tooth Mn was lower than current capabilities for measuring high-resolution exposure data in tooth biomarkers,^7,44^ our use of the tooth biomarker nonetheless allowed us to quantify prospective associations of Mn exposure with neurodevelopment in three distinct exposure periods. Because Mn is both an essential nutrient and neurotoxicant, understanding how its toxicity may differ in the prenatal, postnatal, and childhood periods is essential for public health interventions. Further, teeth have other benefits over traditional exposure markers (e.g., blood) because they provide an objective, noninvasive, integrated measure of exposure across multiple exposure sources (e.g., air, water, and soil), which is particularly important for our study population, where children were likely exposed to Mn from multiple sources due to their residential proximity to ferroalloy industry.^63^

## Conclusion

In conclusion, we found suggestive evidence that prenatal and postnatal tooth Mn is protective for some attention-related behaviors in adolescence, particularly inattention, which was not observed for Mn levels in childhood. These findings suggest that the timing of exposure is critical when considering the neurotoxicity of Mn in children.

## Conflicts of interest

The authors declare that they have no conflicts of interest with regard to the content of this report.

## Supplementary Material

## References

[1] Li L Yang X. The essential element manganese, oxidative stress, and metabolic diseases: links and interactions. Oxid Med Cell Longev. 2018;2018:7580707. 29849912 10.1155/2018/7580707 PMC5907490

[2] Horning KJ Caito SW Tipps KG Bowman AB Aschner M. Manganese is essential for neuronal health. Annu Rev Nutr. 2015;35:71–108. 25974698 10.1146/annurev-nutr-071714-034419 PMC6525788

[3] Zoni S Lucchini RG. Manganese exposure: cognitive, motor and behavioral effects on children: a review of recent findings. Curr Opin Pediatr. 2013;25:255–260. 23486422 10.1097/MOP.0b013e32835e906b PMC4073890

[4] Amos-Kroohs RM Davenport LL Atanasova N. Developmental manganese neurotoxicity in rats: cognitive deficits in allocentric and egocentric learning and memory. Neurotoxicol Teratol. 2017;59:16–26. 27756629 10.1016/j.ntt.2016.10.005 PMC5235975

[5] Betharia S Maher TJ. Neurobehavioral effects of lead and manganese individually and in combination in developmentally exposed rats. Neurotoxicology. 2012;33:1117–1127. 22732189 10.1016/j.neuro.2012.06.002

[6] Bauer JA White RF Coull BA. Critical windows of susceptibility in the association between manganese and neurocognition in Italian adolescents living near ferro-manganese industry. Neurotoxicology. 2021;87:51–61. 34478771 10.1016/j.neuro.2021.08.014 PMC8595706

[7] Horton MK Hsu L Henn BC. Dentine biomarkers of prenatal and early childhood exposure to manganese, zinc and lead and childhood behavior. Environ Int. 2018;121:148–158. 30205321 10.1016/j.envint.2018.08.045 PMC6373872

[8] Skogheim TS Weyde KVF Engel SM. Metal and essential element concentrations during pregnancy and associations with autism spectrum disorder and attention-deficit/hyperactivity disorder in children. Environ Int. 2021;152:106468. 33765546 10.1016/j.envint.2021.106468

[9] Hurley LS. The roles of trace elements in foetal and neonatal development. Philos Trans R Soc Lond B Biol Sci. 1981;294:145–152. 6118892 10.1098/rstb.1981.0095

[10] Yoon M Nong A Clewell HJ Taylor MD Dorman DC Andersen ME. Evaluating placental transfer and tissue concentrations of manganese in the pregnant rat and fetuses after inhalation exposures with a PBPK model. Toxicol Sci. 2009;112:44–58. 19726578 10.1093/toxsci/kfp198

[11] Mistry HD Williams PJ. The importance of antioxidant micronutrients in pregnancy. Oxid Med Cell Longev. 2011;2011:841749. 21918714 10.1155/2011/841749 PMC3171895

[12] Chung SE Cheong HK Ha EH. Maternal blood manganese and early neurodevelopment: the mothers and children’s environmental health (MOCEH) study. Environ Health Perspect. 2015;123:717–722. 25734517 10.1289/ehp.1307865 PMC4492260

[13] Lin CC Chen YC Su FC. In utero exposure to environmental lead and manganese and neurodevelopment at 2 years of age. Environ Res. 2013;123:52–57. 23578827 10.1016/j.envres.2013.03.003

[14] Yu XD Zhang J Yan CH Shen XM. Prenatal exposure to manganese at environment relevant level and neonatal neurobehavioral development. Environ Res. 2014;133:232–238. 24971720 10.1016/j.envres.2014.04.012

[15] Bauer JA Claus Henn B Austin C. Manganese in teeth and neurobehavior: sex-specific windows of susceptibility. Environ Int. 2017;108:299–308. 28941415 10.1016/j.envint.2017.08.013 PMC5679133

[16] Mora AM Arora M Harley KG. Prenatal and postnatal manganese teeth levels and neurodevelopment at 7, 9, and 10.5 years in the CHAMACOS cohort. Environ Int. 2015;84:39–54. 26209874 10.1016/j.envint.2015.07.009 PMC4570875

[17] Hong SB Kim JW Choi BS. Blood manganese levels in relation to comorbid behavioral and emotional problems in children with attention-deficit/hyperactivity disorder. Psychiatry Res. 2014;220:418–425. 25064383 10.1016/j.psychres.2014.05.049

[18] Carvalho CF Menezes-Filho JA de Matos VP. Elevated airborne manganese and low executive function in school-aged children in Brazil. Neurotoxicology. 2014;45:301–308. 24308913 10.1016/j.neuro.2013.11.006

[19] Bhang SY Cho SC Kim JW. Relationship between blood manganese levels and children’s attention, cognition, behavior, and academic performance-a nationwide cross-sectional study. Environ Res. 2013;126:9–16. 23790803 10.1016/j.envres.2013.05.006

[20] Menezes-Filho JA de Carvalho-Vivas CF Viana GFS. Elevated manganese exposure and school-aged children’s behavior: a gender-stratified analysis. Neurotoxicology. 2014;45:293–300. 24121006 10.1016/j.neuro.2013.09.006

[21] Rodrigues JLG Araújo CFS Dos Santos NR. Airborne manganese exposure and neurobehavior in school-aged children living near a ferro-manganese alloy plant. Environ Res. 2018;167:66–77. 30007874 10.1016/j.envres.2018.07.007

[22] Sears L Myers JV Sears CG Brock GN Zhang C Zierold KM. Manganese body burden in children is associated with reduced visual motor and attention skills. Neurotoxicol Teratol. 2021;88:107021. 34428495 10.1016/j.ntt.2021.107021 PMC8578377

[23] Soetrisno FN Delgado-Saborit JM. Chronic exposure to heavy metals from informal e-waste recycling plants and children’s attention, executive function and academic performance. Sci Total Environ. 2020;717:137099. 32092800 10.1016/j.scitotenv.2020.137099

[24] Khan K Factor-Litvak P Wasserman GA. Manganese exposure from drinking water and children’s classroom behavior in Bangladesh. Environ Health Perspect. 2011;119:1501–1506. 21493178 10.1289/ehp.1003397 PMC3230445

[25] Bouchard M Laforest F Vandelac L Bellinger D Mergler D. Hair manganese and hyperactive behaviors: pilot study of school-age children exposed through tap water. Environ Health Perspect. 2007;115:122–127. 10.1289/ehp.9504 PMC1797845 17366831

[26] Oulhote Y Mergler D Barbeau B. Neurobehavioral function in school-age children exposed to manganese in drinking water. Environ Health Perspect. 2014;122:1343–1350. 25260096 10.1289/ehp.1307918 PMC4256698

[27] Nascimento S Baierle M Göethel G. Associations among environmental exposure to manganese, neuropsychological performance, oxidative damage and kidney biomarkers in children. Environ Res. 2016;147:32–43. 26844420 10.1016/j.envres.2016.01.035

[28] He B Wang Y Li S. A cross–sectional survey of preschool children: exploring heavy metal exposure, neurotransmitters, and neurobehavioural relationships and mediation effects. Ecotoxicol Environ Saf. 2021;220:112391. 34090107 10.1016/j.ecoenv.2021.112391

[29] Carvalho CF Oulhote Y Martorelli M. Environmental manganese exposure and associations with memory, executive functions, and hyperactivity in Brazilian children. Neurotoxicology. 2018;69:253–259. 29432852 10.1016/j.neuro.2018.02.002

[30] Kern CH Stanwood GD Smith DR. Pre-weaning manganese exposure causes hyperactivity, disinhibition, and spatial learning and memory deficits associated with altered dopamine receptor and transporter levels. Synapse. 2010;64:363–378. 20029834 10.1002/syn.20736 PMC2840192

[31] Beaudin SA Strupp BJ Strawderman M Smith DR. Early postnatal manganese exposure causes lasting impairment of selective and focused attention and arousal regulation in adult rats. Environ Health Perspect. 2017;125:230–237. 27384154 10.1289/EHP258 PMC5289906

[32] Lasley SM Fornal CA Mandal S Strupp BJ Beaudin SA Smith DR. Early postnatal manganese exposure reduces rat cortical and striatal biogenic amine activity in adulthood. Toxicol Sci. 2020;173:144–155. 31560393 10.1093/toxsci/kfz208 PMC6944216

[33] Conley TE Beaudin SA Lasley SM. Early postnatal manganese exposure causes arousal dysregulation and lasting hypofunctioning of the prefrontal cortex catecholaminergic systems. J Neurochem. 2020;153:631–649. 31811785 10.1111/jnc.14934 PMC7261255

[34] Neal AP Guilarte TR. Mechanisms of lead and manganese neurotoxicity. Toxicol Res (Camb). 2013;2:99–114. 25722848 10.1039/C2TX20064C PMC4338437

[35] Lucchini R Placidi D Cagna G. Manganese and developmental neurotoxicity. Adv Neurobiol. 2017;18:13–34. 28889261 10.1007/978-3-319-60189-2_2 PMC6057616

[36] Arnsten AFT. The emerging neurobiology of attention deficit hyperactivity disorder: the key role of the prefrontal association cortex. J Pediatr. 2009;154:I.S43. 10.1016/j.jpeds.2009.01.018 PMC2894421 20596295

[37] Kreutzer JS DeLuca J Caplan B, eds. Encyclopedia of Clinical Neuropsychology. Springer; 2011.

[38] Jangmo A Stålhandske A Chang Z. Attention-Deficit/Hyperactivity disorder, school performance, and effect of medication. J Am Acad Child Adolesc Psychiatry. 2019;58:423–432. 30768391 10.1016/j.jaac.2018.11.014 PMC6541488

[39] Commodari E Guarnera M. Attention and reading skills. Percept Mot Skills. 2005;100:375–386. 15974348 10.2466/pms.100.2.375-386

[40] Greenfield EA Moorman S Rieger A. Life course pathways from childhood socioeconomic status to later-life cognition: evidence from the wisconsin longitudinal study. J Gerontol B Psychol Sci Soc Sci. 2021;76:1206–1217. 32369603 10.1093/geronb/gbaa062 PMC8200350

[41] Acacio-Claro PJ Doku DT Koivusilta LK Rimpelä AH. How socioeconomic circumstances, school achievement and reserve capacity in adolescence predict adult education level: a three-generation study in Finland. Int J Adolesc Youth. 2017;23:382–397.

[42] Chiu YM Claus Henn B Hsu HL. Sex differences in sensitivity to prenatal and early childhood manganese exposure on neuromotor function in adolescents. Environ Res. 2017;159:458–465. 28858760 10.1016/j.envres.2017.08.035 PMC5623637

[43] Schildroth S Friedman A White RF. Associations of an industry-relevant metal mixture with verbal learning and memory in Italian adolescents: the modifying role of iron status. Environ Res. 2023;224:115457. 36773645 10.1016/j.envres.2023.115457 PMC10117691

[44] Claus Henn B Austin C Coull BA. Uncovering neurodevelopmental windows of susceptibility to manganese exposure using dentine microspatial analyses. Environ Res. 2018;161:588–598. 29247915 10.1016/j.envres.2017.12.003 PMC5965684

[45] National Longitudinal Surveys. Appendix A: HOME-SF Scales (NLSY79 Child). Published online 1979.

[46] Lucchini RG Guazzetti S Zoni S. Tremor, olfactory and motor changes in Italian adolescents exposed to historical ferro-manganese emission. Neurotoxicology. 2012;33:687–696. 22322213 10.1016/j.neuro.2012.01.005 PMC3360122

[47] Arora M Hare D Austin C Smith DR Doble P. Spatial distribution of manganese in enamel and coronal dentine of human primary teeth. Sci Total Environ. 2011;409:1315–1319. 21211818 10.1016/j.scitotenv.2010.12.018

[48] Arora M Bradman A Austin C. Determining fetal manganese exposure from mantle dentine of deciduous teeth. Environ Sci Technol. 2012;46:5118–5125. 22455722 10.1021/es203569f PMC3341525

[49] Sabel N Johansson C Kühnisch J. Neonatal lines in the enamel of primary teeth-a morphological and scanning electron microscopic investigation. Arch Oral Biol. 2008;53:954–963. 18589400 10.1016/j.archoralbio.2008.05.003

[50] Evans AS Preston A. Conners Rating Scales. Springer; 2011.

[51] Keith Conners C Sitarenios G Parker JDA Epstein JN. Revision and restandardization of the Conners Teacher Rating Scale (CTRS-R): factor structure, reliability, and criterion validity. J Abnorm Child Psychol. 1998;26:279–291. 9700520 10.1023/a:1022606501530

[52] Su CT Wang WC Shur-Fen Gau S. Examination of the psychometric properties of the conners’ parent and teacher rating scale-revised short form using multidimensional rasch modeling. Taiwan J Psychiatry. 2009;23:146–157.

[53] Green M Wong M Atkins D Taylor J Feinleib M. Diagnosis of Attention-Deficit/Hyperactivity Disorder. Agency for Health Care Policy and Research (US). 3; 1999. 20734519

[54] Lucchini RG Zoni S Guazzetti S. Inverse association of intellectual function with very low blood lead but not with manganese exposure in Italian adolescents. Environ Res. 2012;118:65–71. 22925625 10.1016/j.envres.2012.08.003 PMC3477579

[55] Cesana GC Ferrario M De Vito G Sega R Grieco A. Evaluation of the socioeconomic status in epidemiological surveys: hypotheses of research in the brianza area MONICA project. Med Lav. 1995;86:16–26. 7791660

[56] Eastman RR Jursa TP Benedetti C Lucchini RG Smith DR. Hair as a biomarker of environmental manganese exposure. Environ Sci Technol. 2013;47:1629–1637. 23259818 10.1021/es3035297 PMC3583582

[57] Smith D Gwiazda R Bowler R. Biomarkers of Mn exposure in humans. Am J Ind Med. 2007;50:801–811. 17924418 10.1002/ajim.20506

[58] Claus Henn B Ettinger AS Schwartz J. Early postnatal blood manganese levels and children’s neurodevelopment. Epidemiology. 2010;21:433–439. 20549838 10.1097/ede.0b013e3181df8e52 PMC3127440

[59] Sanders T Liu Y Buchner V Tchounwou PB. Neurotoxic effects and biomarkers of lead exposure: a review. Rev Environ Health. 2009;24:15–45. 19476290 10.1515/reveh.2009.24.1.15 PMC2858639

[60] Harel O Mitchell EM Perkins NJ. Multiple imputation for incomplete data in epidemiologic studies. Am J Epidemiol. 2018;187:576–584. 29165547 10.1093/aje/kwx349 PMC5860387

[61] Zhou XH Eckert GJ Tierney WM. Multiple imputation in public health research. Stat Med. 2001;20:1541–1549. 11343373 10.1002/sim.689

[62] Buuren S van Groothuis-Oudshoorn K. MICE: multivariate imputation by chained equations in R. J Stat Softw. 2011;45:1–67.

[63] Butler L Gennings C Peli M. Assessing the contributions of metals in environmental media to exposure biomarkers in a region of ferroalloy industry. J Expo Sci Environ Epidemiol. 2019;29:674–687. 30337680 10.1038/s41370-018-0081-6 PMC6472994

[64] Rubin DB. Multiple Imputation for Nonresponse in Surveys. Wiley; 2004.

[65] Sánchez BN Hu H Litman HJ Téllez-Rojo MM. Statistical methods to study timing of vulnerability with sparsely sampled data on environmental toxicants. Environ Health Perspect. 2011;119:409–415. 21362588 10.1289/ehp.1002453 PMC3060007

[66] Broberg K Taj T Guazzetti S. Manganese transporter genetics and sex modify the association between environmental manganese exposure and neurobehavioral outcomes in children. Environ Int. 2019;130:104908. 31233999 10.1016/j.envint.2019.104908 PMC6682429

[67] Wahlberg K Arora M Curtin A. Polymorphisms in manganese transporters show developmental stage and sex specific associations with manganese concentrations in primary teeth. Neurotoxicology. 2018;64:103–109. 28917719 10.1016/j.neuro.2017.09.003 PMC6053672

[68] Peres TV Schettinger MRC Chen P. Manganese-induced neurotoxicity: a review of its behavioral consequences and neuroprotective strategies. BMC Pharmacol Toxicol. 2016;17:57. 27814772 10.1186/s40360-016-0099-0 PMC5097420

[69] van Dyck LI Morrow EM. Genetic control of postnatal human brain growth. Curr Opin Neurol. 2017;30:114. 27898583 10.1097/WCO.0000000000000405 PMC5340196

[70] Rahman SM Kippler M Tofail F Bölte S Hamadani JD Vahter M. Manganese in drinking water and cognitive abilities and behavior at 10 years of age: a prospective cohort study. Environ Health Perspect. 2017;125. 10.1289/EHP631 PMC5726374 28564632

[71] Schullehner J Thygesen M Kristiansen SM Hansen B Pedersen CB Dalsgaard S. Exposure to manganese in drinking water during childhood and association with attention-deficit hyperactivity disorder: a nationwide cohort study. Environ Health Perspect. 2020;128:1–10. 10.1289/EHP6391 PMC7505135 32955354

[72] Uytun MC. Development Period of Prefrontal Cortex. Prefrontal Cortex. [Published online ahead of print October 3, 2018].

[73] Willard VW Conklin HM Huang L Zhang H Kahalley LS. Concordance of parent-, teacher- and self-report ratings on the conners 3 in adolescent survivors of cancer. Psychol Assess. 2016;28:1110–1118. 27537005 10.1037/pas0000265 PMC4991558

[74] Wochos GC Semerjian CH Walsh KS. Differences in parent and teacher rating of everyday executive function in pediatric brain tumor survivors. Clin Neuropsychol. 2014;28:1243–1257. 25343533 10.1080/13854046.2014.971875

[75] McCann S Amadó MP Moore SE. The role of iron in brain development: a systematic review. Nutrients. 2020;12:1–23. 10.3390/nu12072001 PMC7400887 32635675

[76] Kim Y Park S. Iron deficiency increases blood concentrations of neurotoxic metals in children. Korean J Pediatr. 2014;57:345–350. 25210521 10.3345/kjp.2014.57.8.345 PMC4155178

