# Supplementary material for: Early life manganese exposure and reported attention-related behaviors in Italian adolescents
Source: Environ Epidemiol. 2023 Oct 19;7(6):e274. doi: 10.1097/EE9.0000000000000274 (PMC11189689; doi:10.1097/EE9.0000000000000274)
Supplement: Supplementary file 1 [file ee9-7-e274-s001.docx]

**SUPPLEMENTARY INFORMATION**

**eTable 1.** Number (%) of adolescents missing data for key covariates in this analysis (n= 125).

| Characteristic | N (%) |
| --- | --- |
| Sex | 0 (0) |
| Age | 0 (0) |
| Socioeconomic status index | 1 (1) |
| HOME score | 8 (6) |
| Site | 0 (0) |
| Tooth loss due to attrition | 0 (0) |
| Conners, parent-reported T-scores  ADHD index  Hyperactivity  Inattention  Oppositional behavior | 0 (0)  0 (0)  0 (0)  0 (0) |
| Conners, teacher-reported T-scores  ADHD index  Hyperactivity  Inattention  Oppositional behavior | 0 (0)  0 (0)  0 (0)  0 (0) |
| Conners, self-reported T-scores  ADHD index  Hyperactivity  Inattention  Anger problems  Conduct problems  Emotional problems  Family problems | 0 (0)  0 (0)  0 (0)  0 (0)  0 (0)  0 (0)  0 (0) |
| DSM-IV symptom subscales T-scores  Impulsivity  Disattention  Total problems | 0 (0)  0 (0)  0 (0) |
| Metal biomarkers  Prenatal Mn (AUC ^55^Mn:Ca^43^ x 10^4^)  Postnatal Mn (AUC ^55^Mn:Ca^43^ x 10^4^)  Childhood Mn (^55^Mn:Ca^43^)  Blood Pb (µg/dL) | 0 (0)  0 (0)  0 (0)  3 (2) |

*ADHD= attention deficit hyperactivity disorder, HOME= Home Observation Measurement of the Environment, AUC= area under curve, Mn= manganese, Ca= calcium, Pb= lead.

**eTable 2.** Summary statistics for complete and multiple imputed data in PHIME.

| Characteristic | Imputed data, N (%) or mean (SD) | Complete data, N (%) or mean (SD) |
| --- | --- | --- |
| Sex  Female  Male | 68 (54.4%)  57 (45.6%) | 68 (54.4%)  57 (45.6%) |
| Age (years) | 11.9 (0.9) | 11.9 (0.9) |
| Socioeconomic status index  Low  Medium  High | 26 (20.8%)  71 (56.8%)  28 (22.3%) | 26 (20.8%)  70 (56.8%)  28 (22.4%) |
| HOME score | 6.3 (1.4) | 6.2 (1.4) |
| Site  Bagnolo Mella  Garda Lake  Valcamonica | 74 (59.2%)  28 (22.4%)  23 (18.4%) | 74 (59.2%)  28 (22.4%)  23 (18.4%) |
| Tooth loss due to attrition  None  Less than one-third  More than one-third | 68 (54.4%)  46 (36.8%)  11 (8.8%) | 68 (54.4%)  46 (36.8%)  11 (8.8%) |
| Conners parent-reported T-scores  ADHD index  Hyperactivity  Inattention  Oppositional behavior | 50.1 (11.3)  48.1 (8.2)  48.9 (10.8)  49.1 (10.0) | 50.1 (11.3)  48.1 (8.2)  48.9 (10.8)  49.1 (10.0) |
| Conners teacher-reported T-scores  ADHD index  Hyperactivity  Inattention  Oppositional behavior | 45.8 (6.4)  45.0 (5.1)  47.5 (7.6)  45.4 (4.3) | 45.8 (6.5)  45.0 (5.1)  47.5 (7.6)  45.4 (4.3) |
| Metal biomarkers: median (25^th^, 75^th^ percentile)  Prenatal Mn (AUC ^55^Mn:Ca^43^ x 10^4^)  Postnatal Mn (AUC ^55^Mn:Ca^43^ x 10^4^)  Childhood Mn (^55^Mn:Ca^43^)  Blood Pb (µg/dL) | 0.4 (0.3, 0.5)  0.1 (0.1, 0.02)  0.0006 (0.0005, 0.0009)  1.6 (1.0, 1.8) | 0.4 (0.3, 0.5)  0.1 (0.1, 0.02)  0.0006 (0.0005, 0.0009)  1.6 (1.0, 1.8) |

*ADHD= attention deficit hyperactivity disorder, HOME= Home Observation Measurement of the Environment, AUC= area under curve, Mn= manganese, Ca= calcium, Pb= lead.

**eTable 3.** Beta coefficients (β) and 95% confidence intervals (CIs) from linear regression models quantifying the association between tooth Mn levels measured in the prenatal, postnatal, and childhood periods with parent- and teacher-reported scales from the Conners Rating Scales for the full dataset (n= 125), males (n= 57), and females (n= 68). Beta coefficients reflect the percent change in age- and sex-adjusted Conners T-scores for a doubling in tooth Mn levels. Multivariable linear regression models were mutually adjusted for Mn in all exposure periods, as well as socioeconomic status, HOME score, tooth attrition, and ln-transformed blood Pb.

|  | Conners scale | Prenatal  β (95% CI) | Postnatal  β (95% CI) | Childhood  β (95% CI) |
| --- | --- | --- | --- | --- |
| Full dataset | Parent ADHD index | 1.26 (-6.16, 9.26) | -4.60 (-9.53, 0.59) | 3.60 (-0.94, 8.35) |
|  | Parent hyperactivity | -1.10 (-6.46, 4.56) | -1.10 (-4.92, 2.87) | 1.68 (-1.72, 5.19) |
|  | Parent inattention | 1.68 (-5.51, 9.42) | -4.47 (-9.28, 0.59) | 2.88 (-1.49, 7.45) |
|  | Parent oppositional behavior | 1.33 (-5.71, 8.89) | -1.17 (-6.02, 3.92) | 3.45 (-0.95, 8.05) |
|  | Teacher ADHD index | -1.51 (-6.47, 3.70) | -1.24 (-4.67, 2.31) | -0.14 (-3.21, 3.03) |
|  | Teacher hyperactivity | 1.12 (-2.92, 5.32) | 0.77 (-2.07, 3.68) | -0.48 (-2.89, 1.98) |
|  | Teacher inattention | -5.26 (-10.27, 0.03) | -2.40 (-6.04, 1.39) | -0.42 (-3.61, 2.89) |
|  | Teacher oppositional behavior | 2.03 (-1.38, 5.56) | 0.00 (-2.42, 2.48) | 0.07 (-1.95, 2.13) |
| Males | Parent ADHD index | 1.75 (-6.34, 10.54) | -3.81 (-9.02, 1.70) | 2.53 (-2.90, 8.25) |
|  | Parent hyperactivity | -0.14 (-6.95, 7.17) | -0.14 (-4.78, 4.72) | 3.81 (-1.01, 8.87) |
|  | Parent inattention | 1.12 (-7.05, 10.00) | -2.13 (-7.43, 3.48) | 2.53 (-3.03, 8.40) |
|  | Parent oppositional behavior | 2.60 (-7.97, 14.38) | -3.61 (-10.43, 3.73) | 2.67 (-4.59, 10.48) |
|  | Teacher ADHD index | -4.21 (-12.06, 4.35) | -1.99 (-7.43, 3.77) | -0.35 (-5.87, 5.51) |
|  | Teacher hyperactivity | 1.19 (-5.84, 8.74) | -0.97 (-5.69, 4.00) | -0.97 (-5.56, 3.86) |
|  | Teacher inattention | -9.94 (-16.99, -2.29) | -3.34 (-8.45, 2.06) | -2.53 (-7.69, 2.91) |
|  | Teacher oppositional behavior | 2.17 (-2.57, 7.15) | -2.60 (-5.60, 0.49) | -0.55 (-3.61, 2.60) |
| Females | Parent ADHD index | 3.38 (-11.21, 20.37) | -8.87 (-19.25, 2.84) | 4.61 (-3.19, 13.03) |
|  | Parent hyperactivity | -1.31 (-10.87, 9.28) | -5.98 (-13.22, 1.86) | 1.40 (-3.71, 6.77) |
|  | Parent inattention | 6.36 (-7.40, 22.17) | -12.34 (-21.47, -2.14) | 4.10 (-3.00, 11.72) |
|  | Parent oppositional behavior | 0.14 (-10.66, 12.24) | 1.12 (-7.56, 10.60) | 2.46 (-3.36, 8.62) |
|  | Teacher ADHD index | 4.54 (-2.20, 11.73) | -3.21 (-8.20, 2.06) | -0.90 (-4.21, 2.53) |
|  | Teacher hyperactivity | 1.82 (-2.51, 6.34) | 0.14 (-3.34, 3.74) | -0.83 (-2.96, 1.35) |
|  | Teacher inattention | 4.83 (-3.51, 13.88) | -5.26 (-11.24, 1.12) | 0.63 (-3.52, 4.95) |
|  | Teacher oppositional behavior | 3.38 (-2.09, 9.16) | 0.70 (-3.46, 5.03) | -0.55 (-3.22, 2.19) |

*ADHD= attention deficit hyperactivity disorder.

**eTable 4.** P-values from multiple informant models comparing associations between a doubling in tooth Mn levels measured in the prenatal, postnatal, and childhood periods with parent- and teacher-reported scales from the Conners Rating Scales.

| Conners Scale | p-value |
| --- | --- |
| Parent-reported |  |
| ADHD index | 0.10 |
| Hyperactivity | 0.52 |
| Inattention | 0.15 |
| Oppositional | 0.40 |
| Teacher-reported |  |
| ADHD index | 0.51 |
| Hyperactivity | 0.67 |
| Inattention | **0.01** |
| Oppositional | 0.76 |

*ADHD= attention deficit hyperactivity disorder.

**eTable 5.** P-values from multiple informant models comparing associations between a doubling in tooth Mn levels measured in the prenatal, postnatal, and childhood periods with parent- and teacher-reported scales from the Conners Rating Scales from sex-stratified models.

| Conners Scale | Male p-value | Female p-value |
| --- | --- | --- |
| Parent-reported |  |  |
| ADHD index | 0.12 | 0.45 |
| Hyperactivity | 0.29 | 0.20 |
| Inattention | 0.33 | 0.20 |
| Oppositional | 0.56 | 0.66 |
| Teacher-reported |  |  |
| ADHD index | 0.35 | 0.46 |
| Hyperactivity | 0.87 | 0.80 |
| Inattention | **0.004** | 0.61 |
| Oppositional | 0.61 | 0.33 |

*ADHD= attention deficit hyperactivity disorder.

**eFigure 1.** Directed acyclic graph (DAG) reflecting hypothesized associations of covariates in the Public Health Impact of Metals Exposure (PHIME) study.


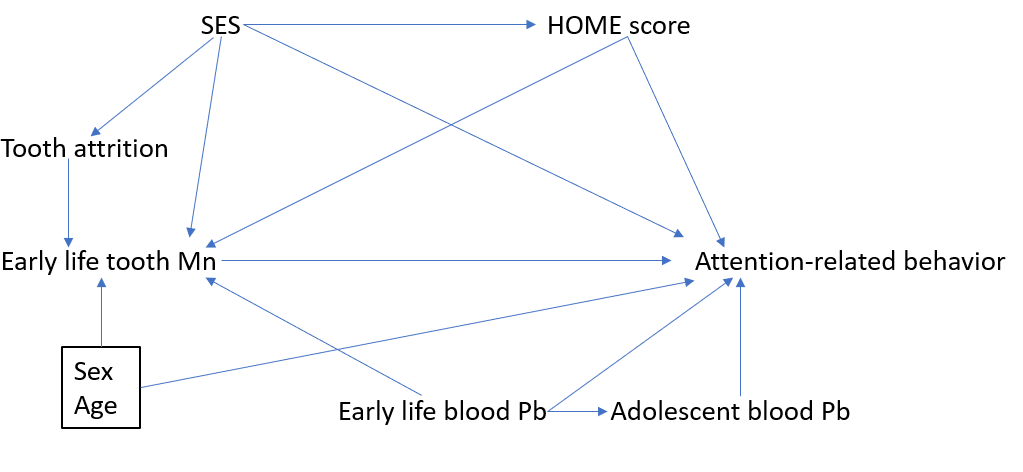


*HOME= Home Observation Measurement of the Environment, SES= socioeconomic status, Mn= manganese, Pb= lead.

**Age and sex were not included as covariates in the analysis because Conners scores were age- and sex-standardized.
